# Do anti-amyloid beta protein antibody cross reactivities confound Alzheimer disease research?

**DOI:** 10.1186/s12952-017-0066-3

**Published:** 2017-01-26

**Authors:** Sally Hunter, Carol Brayne

**Affiliations:** Department of Public Health and Primary Care, Institute of Public Health Forvie Site, University of Cambridge School of Clinical Medicine, Box 113 Cambridge Biomedical Campus, Cambridge, CB2 0SP UK

**Keywords:** Alzheimer disease, Amyloid beta protein, Amyloid precursor protein, Antibody, Cross reactivity, Experimental design

## Abstract

**Background:**

Alzheimer disease (AD) research has focussed mainly on the amyloid beta protein (Aβ). However, many Aβ-and P3-type peptides derived from the amyloid precursor protein (APP) and peptides thought to derive from Aβ catabolism share sequence homology. Additionally, conformations can change dependent on aggregation state and solubility leading to significant uncertainty relating to interpretations of immunoreactivity with antibodies raised against Aβ. We review evidence relating to the reactivities of commonly used antibodies including 6F3D, 6E10 and 4G8 and evaluate their reactivity profiles with respect to AD diagnosis and research.

**Results:**

Antibody cross-reactivities between Aβ-type, P3-type and Aβ-catabolic peptides confound interpretations of immunoreactivity. More than one antibody is required to adequately characterise Aβ. The relationships between anti-Aβ immunoreactivity, neuropathology and proposed APP cleavages are unclear.

**Conclusions:**

We find that the concept of Aβ lacks clarity as a specific entity. Anti-Aβ antibody cross-reactivities lead to significant uncertainty in our understanding of the APP proteolytic system and its role in AD with profound implications for current research and therapeutic strategies.

## Introduction

**Fig. 1:**
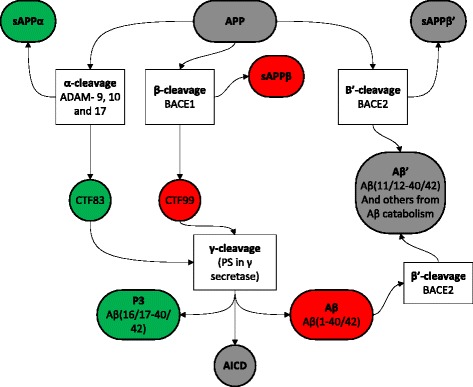
APP cleavage pathways. Green: sequential α- and γ- cleavages of the α- pathway, red: sequential β- and γ- cleavages of the β- pathway, grey: alternative fragments from β’ cleavage or shared full length APP and AICD. Other cleavage pathways such as δ and η are not shown

Research into the causes and progression pathways of Alzheimer disease (AD) has focussed primarily on the roles of the amyloid beta protein (Aβ) derived from the amyloid precursor protein (APP) via sequential proteolytic cleavages [1, 2]. In summary, there are two main APP cleavage pathways, Fig. 1. The α-pathway involves an initial α-cleavage to release the large extracellular soluble sAPPα leaving the 83 amino acid (aa) residue carboxy terminal fragment (CTF) in the membrane. This is further processed by γ-secretase containing Presenilin (PS) to release the variable length P3 peptide and the APP intracellular domain (AICD). This pathway is thought to be constitutive and α-cleavage precludes processing by the β-secretase BACE1 as it cuts within the Aβ sequence. In competition with α-cleavage and with APP expression as rate limiting [3], β-cleavage releases the large extracellular soluble sAPPβ leaving a 99 aa residue CTF in the membrane that is further processed by the shared sequential γ-secretase to release the variable length Aβ and the AICD. The main fragments expressed are the large sAPPα and sAPPβ domains, the smaller variable length Aβ and P3 fragments and the AICD, all sharing sequence homology to varying degrees with each other and with full length APP. Additional APP cleavages include β’-cleavage by BACE2 [4], δ- and η-cleavage [5, 6] and cleavage by caspase [7]. BACE2 may also be involved in catabolism of Aβ [8].

Evidence relating to Aβ from autosomal dominant genetic mutations in the amyloid precursor protein (APP) and presenilins (PS) in familial AD (FAD) [9, 10], coupled with the neuropathological diagnostic value associated with the presence of deposits of Aβ in the brain in both FAD and sporadic AD (SAD) [11, 12], has been interpreted in the amyloid cascade hypothesis as showing a causal role for Aβ in disease progression [13, 14] and has been updated to reflect the ratios of Aβ (1–42)/Aβ (1–40) [14, 15] or oligomers [16, 17]. However, this interpretation of the evidence relating to Aβ has not been fully accepted and alternative interpretations including the presenilin hypothesis [18, 19] and the APP matrix approach [20, 21] have been put forward.

In addition to Aβ40 and Aβ42, the peptides at the main focus of research, there are many soluble [22] and insoluble Aβ-type peptides, including N-terminal extended peptides [23], that have yet to be fully described and accounted for in theoretical and experimental disease models. In addition to different sequences, Aβ-type peptides can exist in a variety of aggregation states including monomers, dimers, oligomers and fibrils. Evidence that behaviour profiles differ between the various Aβ-type sequences and aggregation states suggests that some Aβ species, such as Aβ42 or oligomers, may be more important in disease progression than others.

Evidence from population studies [24–26] suggests that correspondence between clinical dementia status and neuropathological diagnosis blind to clinical dementia status in the older population where most dementia occurs, do not correspond well. The relationships between Aβ, neuropathology and clinical dementia status are not clear. In order to investigate these relationships an understanding of the different presentations of Aβ across the different sequence lengths, aggregation states and neuropathological associations is required.

AD research has depended greatly on the use of antibodies. Concerns regarding the interpretation and reliability of antibodies relating to reproducibility of science in general have been previously highlighted [27]. Antibodies have been raised against various Aβ epitopes and these recognise slightly different pathological profiles [28–31]. Because Aβ-type peptides share sequence homology and conformations to varying degrees, cross reactivity can potentially confound interpretations of immunoreactivity. Here we look at evidence relating to the reactivities of the commonly used antibodies 6F3D, 6E10 and 4G8 immunoreactive with Aβ and ask how the reactivity profiles of commonly used antibodies relate to AD diagnosis and research.

## Antibody reactivities with peptides from α-, β- and γ- cleavages

**Fig. 2:**
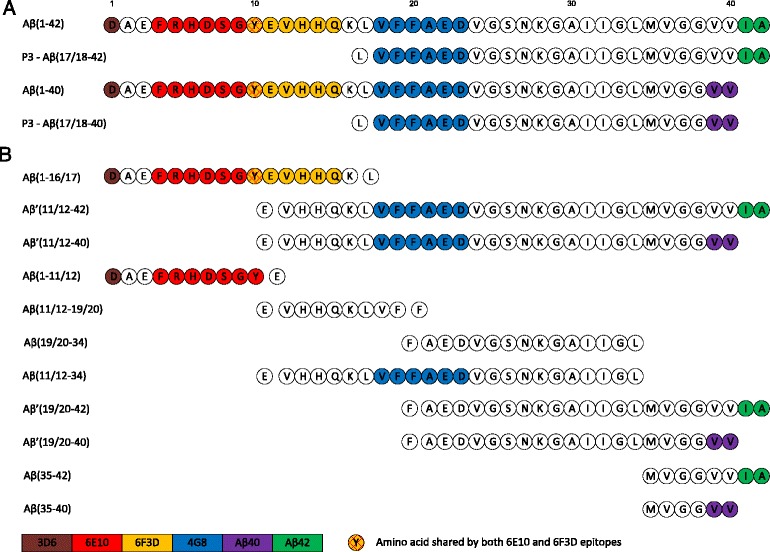
Epitopes recognised by commonly used antibodies in various Aβ –type peptides. **a** fragments associated with the main α- and β- cleavage pathways, **b** fragments associated with BACE2 catabolism. Note: Aβ can exist as monomers, dimers, oligomers and fibrils; epitopes may be lost due to conformational change due to aggregation/solubility etc.; antibodies do not react with specific Aβ sequences in all conditions; amino acids of epitopes for MBC40 (Aβ40) and MBC42 (Aβ42) not well described

The epitopes recognised by 6F3D, 6E10 and 4G8 to various forms of Aβ, Fig. 2a and Table 1, are usually interpreted to be sequence specific and relate to proteolytic fragments released following sequential β- and γ-cleavages.

**Table 1:** Epitopes and cross reactivities of selected antibodies raised against Aβ

Antibody	Epitope	Cross Reactivity	Ref
4G8	Raised against synthetic peptide Aβ17-24; epitope lies within aa 18–23; recognises multiple forms of Aβ	Cross reacts with APP770 and P3; reacts with conformational epitope of aggregated fibrils including α-synuclein	[29, 38, 39]
6E10	Raised against Aβ1-17; epitope lies within aa 4–9; recognises Aβ with intact N-terminal epitope	Cross reacts with APP and Aβ (1–16); No reaction predicted with P3	[3, 29]
6F3D	Raised against synthetic peptide Aβ8-17; epitope lies within aa 10–15; recognises Aβ with intact N-terminal epitope	Predicted to react with Aβ (1–16); Does not react with P3	[29, 31]
MBC40 (Aβ40)	Recognises C-terminal Aβ peptides ending at aa40; epitope not well described	Cross reacts with N-terminal truncated peptides including P3	[29]
MBC42 (Aβ42)	Recognises C-terminal Aβ peptides ending at aa42; epitope not well described	Cross reacts with N-terminal truncated peptides including P3	[29]
BS85	Raised against Aβ (25–35); recognises Aβ38, Aβ39, Aβ40, Aβ42 and Aβ43; epitope not well described	Cross reacts with N-terminal truncated peptides including P3	[28]
BC05	Raised against Aβ (35–43); recognises Aβ42 and Aβ43; epitope not well described	Cross reacts with N-terminal truncated peptides including P3; does not recognise Aβ40; used in commercial ELISA kits for the detection of Aβ42	[28, 44]
BA27	Raised against Aβ (1–40) Recognises Aβ40; 100-1000x more reactive with Aβ40 than Aβ42 and Aβ43; epitope not well described	Cross reacts with N-terminal truncated peptides including P3; used in commercial ELISA kits for the detection of Aβ40	[28]
AβN17 (Leu)	Raised against P3 (40); recognises P3 (40) and synthetic P3 (42) peptide; epitope not well described	Reactivity with insoluble, aggregated P3 (42) not confirmed	[28, 42, 57]
3D6	Raised against Aβ with N-terminal aspartic acid; epitope not well described	Does not cross react with sAPPs or full length APP; No reaction predicted with P3; parent of Bapineuzumab; epitope not well described	[53]
	Raised against Aβ (13–28); epitope not well described	Solanezumab	[53]

6E10 recognises an epitope in the N-terminal region of both Aβ40 and Aβ42. The 6E10 N-terminal epitope is also recognised in Aβ (1-16/17), a fragment that could reflect catabolism of Aβ [3, 32], or additional processing of the C99 carboxy terminal membrane bound fragment (CTF) following β-cleavage [33]. The fragment Aβ (1-11/12) detected in soluble fractions [34] and generated following catabolism of full length Aβ by BACE2 [8] is predicted to react with 6E10, Fig. 2b, but this has not been investigated.

Antibody 6F3D recognises an N-terminal epitope present in full length Aβ42, Aβ40 and is predicted to react with Aβ (1-16/17) but unlike 6E10, not Aβ (1-11/12). Neither 6F3D nor 6E10 are predicted to react with P3-type peptides, equivalent to Aβ (16/17-40/42) derived from sequential α- and γ- cleavages of APP [1] that lack the amino acid sequence of the epitope. As such 6E10 and 6F3D represent initial β-cleavage but do not inform on C-terminal variability due to carboxypeptidase activities of γ-cleavage [35]. This may be generally applicable to other antibodies recognising N-terminal epitopes, such as 6C6, which also recognise N-terminal epitopes [34]. Interpretation could be further complicated by reactivities with shorter N-terminal peptides derived from full length Aβ by catabolism or additional processing of membrane bound CTF and shorter C-terminal endings, seen in conditioned media from cell culture [22].

Antibodies specific for C-terminals ending at aa Aβ40 or Aβ42 are traditionally interpreted as representing Aβ. However, antibodies specific for Aβ40 MBC40 and Aβ42 MBC40 were noted to react with shorter N-truncated Aβ peptides including P3 (40) and P3 (42) respectively [29]. Antibodies reactive with either Aβ40 or Aβ42 are also predicted to react with shorter peptides from Aβ catabolism, Fig. 2, though this will depend on whether the exact epitope recognised is still present in the shorter sequence. Characterisation of epitopes recognised by antibodies in general is a widely held concern. While studies using antibodies raised against Aβ40 or Aβ42 will monitor the specificity of C-terminal epitopes or cross reactivity with full length APP [36], very few account for N-terminal variation. Therefore we cannot be certain that any antibody thought to represent Aβ40 or Aβ42 derived from sequential β- and γ- cleavages actually represents full length rather than peptides from other cleavages lacking the N-terminal epitopes. Immunoreactivity with the antibodies recognising fragments ending in either aa40 or aa42 of Aβ should be interpreted as representing fragments from γ-cleavage regardless of initial α- or β- cleavages unless cross reactivity checks prove otherwise.

Aβ- type, P3-type, Aβ’, equivalent to Aβ (11/12-4x) released following cleavage by BACE2 and γ-cleavage [4] or following catabolism of the full length Aβ peptide [8], and various fragments derived from catabolism share sequence homology, Fig. 2 and Table 1. In human kidney 293 cell culture medium, approximately 69% of peptides released following γ- cleavage were found to be P3-type, around 20% are Aβ-type and 11% are Aβ’-type [37] however, different expression systems may give different ratios and these ratios may not reflect the levels of expression in the human brain. Shorter fragments in Fig. 2, including Aβ (11/12-19/20), Aβ (19/20-34), Aβ (19/20-42), Aβ (19/20-40), Aβ (35–42) and Aβ (35–40) are predicted from BACE2 catabolism of full length Aβ [8]. These shorter Aβ-type peptide sequences have been largely neglected in AD research and their expression levels in human tissues remains to be fully described. No study to date has systematically characterised all these fragments in the human brain. Aβ (1-4x), P3 (17-4x), Aβ’(11/12-x) and Aβ’(11/12-34) type peptides are predicted to react with the 4G8 antibody, reactive with an epitope within aa 18–23. Therefore 4G8 detects products from γ-cleavage irrespective of the initial primary cleavage but should not detect catabolic fragments following degradation of full length Aβ (1-4x) missing the intact epitope sequence. 4G8 also shares a conformational epitope with other fibril forming proteins including α-synuclein [38] that is sequence independent.

While these epitopes are assumed to be sequence specific, this cannot be guaranteed. Aβ exists in many aggregation states from monomers, dimers, oligomers and fibrils. These conformation changes can potentially lead to changes in the presentation of epitopes and neither 6E10 nor 4G8 were found to react with all samples of Aβ when aggregated under various conditions [39]. Reactivity was found to change depending aggregation, suggesting that epitopes can be revealed or hidden by different conformations and at least two different aggregated conformations may be present depending on specific conditions. This study shows that antibodies reactive with Aβ-type peptides are both sequence and conformation dependent [39]. 4G8 specifically was found not to react with Aβ40 at higher molecular weight oligomers and reacted with high molecular weight Aβ42 only when aggregated under conditions with agitation. Therefore 4G8 immunoreactivity cannot be assumed to visualise “total” Aβ.

## The use of antibodies in diagnosis and research

AD diagnosis and research in the human brain depends on the use of antibodies reactive with Aβ. It is essential that results from studies across the world are comparable and attempts to standardise inter-laboratory comparisons of amyloid pathology [30, 40] have found that immunohistochemical approaches are more reliable than silver stain based techniques. Further, because 4G8 shows more Aβ immunoreactive pathology than either 6E10 or 6F3D, it has been recommended as the antibody of choice for diagnostic work to visualise deposits of Aβ [30], perhaps implying that increased immunoreactivity represents increased sensitivity for Aβ. However, if we consider the reactivity of 4G8 with fragments from the wider APP proteolytic system, not all reactivity necessarily represents Aβ. Thal et al. [29] stained sequential sections from a case with extensive Aβ pathology with 6E10, 6F3D, 4G8, MBC40 and MBC42 Table 1. They found strong reactivity of plaques with MBC42 and 4G8 but little reactivity with MBC40 and 6F3D suggesting that the majority of staining was due to N-truncated peptides equivalent to P3 (Aβ17-42) [29]. However, this interpretation of the staining patterns is not straightforward as loss of staining with 6F3D could also reflect a change in aggregation state that hides the 6F3D epitope especially for Aβ42 which may be more prone to aggregation and insolubility or may be lost due to membrane binding [41]. Therefore antibodies to N-terminal epitopes of Aβ, such as 6F3D and 6E10 may not be revealing all Aβ (1-4x).

Over 40 soluble Aβ-type peptides are biologically present [22]. Interestingly, the peptide P3 (42), representing the peptide thought to be associated with neuropathology of diffuse senile plaques [29] is not listed in Table 2 in Wang et al 1996 [22] even though the use of 4G8 as a capture antibody is predicted to react with it. This suggests that the more insoluble P3 (42) aggregated in plaques could adopt a significantly different conformation to P3 (40) found in the soluble compartment and this requires clarification.

The results obtained by Thal et al. [29, 31] are compatible with those using different monoclonal antibodies, BS85 reacting with multiple forms of Aβ, BC05 – reacting with Aβ42/3 and BA27- reacting with Aβ40 in a different study that did not account for N-terminal variation [28]. This study found similar reactivity profiles for BS85 and BC05, marking multiple cored and fleecy amyloid senile plaques whereas BA27, reactive for C-terminal Aβ40, detected cored senile plaques only. In a follow-up study, Iwatsubo et al. [42] used an antibody raised against the N-terminal of P3 to measure the deposition of P3 and found little reactivity, suggesting that P3 is not involved in neuropathological deposition. However as with Aβ42, this could reflect the different solubilities and aggregation states of P3-type peptides where P3 (40) is seen in the soluble pool of fragments whereas P3 (42) is not [22]. It is important to note that synthetic peptides of soluble P3 (40) were used to select the N-terminal antibody AβN17 (Leu) in this study and although reactivity was noted with P3 (42) in western blot analysis, the aggregation of the synthetic P3 (42) peptide was not considered. As with the reactivity of 6F3D with Aβ40 discussed above, the contributions of aggregation state of P3 (42) and consequent loss of epitope cannot be dismissed. Indeed these staining patterns may indicate that the epitopes contained in the N-terminal of P3 are solubility dependent and are lost in aggregated P3 (42), associated with diffuse amyloid deposition and therefore no reactivity in diffuse plaques with antibodies recognising P3 (40) or non-aggregated P3 (42) would be expected. This interpretation is compatible with the scant reactivity of MBC40 and BA27, showing no reactivity for peptides ending with aa40 [29, 42]. Consideration must be given to solubility and aggregation state when interpreting antibody reactivities.

A few studies explicitly account for antibody cross reactivities. Citron et al. [43] describe the reactivity of R1280 as “*a polyclonal antiserum raised against synthetic Aβ*_*1–40*_ *… and precipitates Aβ (4 kDa), p3 (3 kDa), and small amounts of APP*_*S*_” and in another paper [44] they explicitly describe the reactivities of the monoclonal antibody 21 F12 as immunoprecipitating Aβ (42) and P3 (42), the monoclonal antibody BCO5 detecting Aβ (42) and P3 (42) and the polyclonal antibody C42 detecting Aβ (42) and P3 (42). Watson et al. [45] note that both polyclonal R1280 and R1282 immunoprecipitate soluble Aβ, P3 and related peptides. As already discussed Thal et al. [29] explicitly describe the reactivity of MBC40 with Aβ40 and P3 (40) and MBC42 with Aβ42 and P3 (42). However, few more recent studies explicitly account for antibody reactivity profiles with a potential for cross reactivities to confound interpretations of immunoreactivity patterns.

Study designs using a capture antibody reactive with epitopes within the first 16 amino acids of Aβ to select only Aβ peptides resulting from β-cleavage as an initial step e.g. Moore et al. [3, 46] leave any P3 type peptides unrecorded and not accounted for. Where studies use a capture antibody reactive with the N-terminal of Aβ, further characterisation with antibodies detecting C-terminal aa 40 or 42 can be interpreted as representing Aβ40 or Aβ42 from β-cleavage. However, a study using this approach to quantify Aβ40/42 in wet tissues then investigated location using only antibodies reactive with Aβ42 in formalin fixed, paraffin embedded tissues [34] where results have been interpreted as showing both quantity and location of Aβ42. However, this study design is potentially confounded by cross reactivity with P3 (42) in formalin fixed, paraffin embedded tissue that has not been checked. Keeping experimental approaches consistent both within and between studies is a challenge but one that requires urgent attention. Some commercial ELISA kits use BA27 to detect Aβ40 and BC05 to detect Aβ42, however, since both these antibodies are known to also recognise P3 (40) and P3 (42) respectively, we cannot be certain that any results obtained are not confounded by P3. Antibody reactivity profiles with potentially similar peptides from the APP proteolytic system should always be checked.

## How do we best interpret the available evidence derived from antibody reactivities?

Interpretation of the reactivities of antibodies immunoreactive with Aβ-type peptides is not straightforward and is compounded by the lack of systematic definitions of Aβ-type peptides. On the one hand Aβ is often discussed as a homogenous whole, where the different sequence lengths and aggregation states are collapsed under “Aβ” as an umbrella concept. Yet, because the different fragments sequences and aggregation states show discrete behaviours, this umbrella concept may not be useful for more detailed research questions investigating the role (s) of Aβ in disease pathways. Should each possible fragment derived from α-, β-, and γ- cleavages and Aβ catabolism be experimentally controlled for in a systematic approach? The different behaviours of the Aβ-type and P3-type fragments depending on aggregation state suggest that this may be an important issue that has yet to be fully incorporated in experimental design. This is not straightforward as the contributions of each possible sequence can potentially vary with solubility and aggregation state, certainly increasing experimental costs as each fragment is controlled for.

Because 4G8 is increasingly recommended for diagnostic work [30], and because reactivity is interpreted as Aβ (umbrella concept) it is probable that the contributions of P3 (42) to neuropathological classifications have been hidden in current experimental designs and therefore neglected. However, not all laboratories use 4G8 and instead use 6E10 or 6F3D, specific for N-terminal epitopes of Aβ that do not detect P3-type fragments. These antibodies visualise qualitatively different aspects of Aβ deposition, i.e. lacking contributions from P3-type fragments [31] potentially confounding comparisons between studies from different laboratories using different antibodies [29, 30]. If all laboratories were to use 4G8, this would potentially confound how we understand the deposition of specific Aβ-species as it detects a wide range of fragments, not all necessarily Aβ. This confounding would also be relevant to the use of antibodies reactive with The C-terminal residues from Aβ40 and Aβ42, as N-terminal variation is not detected. The only option to systematically detect specific peptides is to use multiple antibodies reacting with the different epitopes or use a capture antibody relevant to the experimental design, such as 4G8 as in [22] or 6E10 as in [3, 22] and then analyse any fragments further with for example, mass spectroscopy. However, it must be born in mind that a single antibody will not capture all possible Aβ- or P3 type fragments in all aggregations states [39] and this must be explicitly accounted for in any experimental design. The different antigen retrieval methods [47], different profiles of Aβ-type fragments in soluble [22] and insoluble fractions and potential loss of epitopes due to aggregation state [39] add further difficulties in systematically characterising Aβ. A “panel” of antibodies to consistently and reliably characterise Aβ-type, P3-type and catabolic fragments in all aggregation states (monomer, dimer, oligomer and fibril) is not currently possible.

## Antibody reactivities and their relevance to APP proteolytic pathways and disease

Contrary to our current understanding, the immunoreactivity profiles of commonly used antibodies do not correspond directly to APP cleavage pathways [1, 2, 8], summarised in Fig. 1. Immunoreactivity of antibodies predicted to have a wide reactivity profile such as 4G8 and potentially those that react with C-terminal epitopes representing aa40 or aa42 cannot be interpreted as giving evidence for initial α- or β- cleavages. Given that antibodies are central to AD research and biomarker development, it is not clear whether the antibodies currently being used to identify C-terminals do indeed reflect Aβ40/42 or whether signals are confounded by P3 (40/42). Additionally, very little account is taken of the different soluble and insoluble compartments, therefore P3 (42), present in neuropathological deposits, may not be present in soluble fractions and can be easily missed if only soluble fractions are investigated. How then do we best approach the search for reliable biomarkers for AD?

Various morphologies of Aβ deposits have been noted and these differ in their immunoreactivity profiles [48] – how immunoreactivity differences associate with the different pathological morphologies have not been systematically investigated with respect to clinical dementia status. The insoluble fragment P3 (42) may be a major constituent of diffuse amyloid deposition and may be relevant to disease pathways however, current approaches have almost completely neglected any contributions it might have. Cross reactivity of commonly used antibodies between the Aβ, P3 type and catabolic peptides confounds our current understanding and may in part explain why clinical and neuropathological diagnoses of AD do not correspond well in the older population. To what extent a lack of understanding of the APP proteolytic system as a whole derives from a misunderstanding of antibody cross reactivities requires careful consideration. Neuropathological characterisation of human brain donations, essential to our understanding of AD, requires re-evaluation.

Current immunotherapeutic approaches to target Aβ have used passive humanised antibodies to enhance removal of Aβ from the brain with the aim of slowing or halting the progression of AD [49]. To date these have had little success [49–52]. Bapineuzumab, Table 1, is based on the monoclonal antibody 3D6 directed towards an N-terminal epitope and Solanezumab, directed at an epitope from the Aβ (13–28) central region [53]. Given the uncertainty surrounding which fragments are responsible for disease progression we highlight here, we have to ask whether antibodies directed only at N-terminal epitopes of Aβ, such as Bapineuzumab, would be expected to change disease course. Following the failure of both Bapineuzumab [51, 52] and Solanezumab [50] in phase III clinical trials, refinements to the therapeutic approach call for earlier, perhaps preventative, use of the antibodies during the prodromal phase of AD, i.e. where a high amyloid signal is seen on MRI but before any cognitive change has occurred. However, the failure of these trials suggests that a return to basic science and a re-evaluation of our current understanding of the role of Aβ in AD is also warranted. How prodromal AD relates to those in the oldest old who have extensive pathology after death but with intact cognitive function in life remains unclear. Clarification of the physiological roles [54–56] of the APP proteolytic system and all its fragments [20, 21] in both in disease and normal ageing in the human population is urgently required.

The implications arising from the cross reactivity of commonly used antibodies to Aβ are profound. Cross reactivity may be hiding more complex relationships between AD and fragments from sequential α-, β- and γ- cleavages that the current favoured model, the amyloid cascade hypothesis, cannot account for. If P3 is indeed involved in disease progression then a more flexible approach to understanding the relationships between all APP proteolytic fragments may be required and both the presenilin hypothesis [18, 19] and the APP matrix approach [20, 21] may be better guides to systematically investigate this complex proteolytic system.

## Conclusions

The concept of Aβ lacks clarity in terms of what we mean by Aβ as a specific biological form and this is further confounded by antibody cross-reactivities. The different solubilities and aggregation states of proteolytic fragments from γ-cleavage and their catabolism add further complexity. These cross reactivities, often over-looked, require urgent attention by the AD research community. More than one antibody is required to adequately characterise Aβ. We do not currently have reliable evidence to identify any specific APP proteolytic fragment as causal in AD progression. The correspondence between Aβ immunoreactivity from any specific antibody, neuropathology and proposed APP cleavages is not clear and may in part explain the lack of correspondence between clinical and neuropathological diagnoses of dementia. These cross reactivities question current therapeutic approaches to reduce Aβ via directed immunotherapies, call for a detailed re-analysis of biomarker results and call into question approaches aimed solely at reducing β-cleavage. A detailed consideration of anti-Aβ antibody cross reactivities reveals significant uncertainty in our current understanding of the APP proteolytic system and how this relates to disease with profound implications for current research and therapeutic strategies.
