# Erratum to: Do anti-amyloid beta protein antibody cross reactivities confound Alzheimer disease research?

**DOI:** 10.1186/s12952-017-0073-4

**Published:** 2017-03-08

**Authors:** Sally Hunter, Carol Brayne

**Affiliations:** Department of Public Health and Primary Care, Institute of Public Health Forvie Site, University of Cambridge School of Clinical Medicine, Box 113 Cambridge Biomedical Campus, Cambridge, CB2 0SP UK

## Erratum

After publication of the original article [1], it came to the authors’ attention that evidence relating to the epitopes recognised and cross reactivities of the antibodies that form the parents of Bapineuzumab and Solanezumab was omitted from Table 1.

**Table 1:** Epitopes and cross reactivities of selected antibodies raised against Aβ

Antibody	Epitope	Cross Reactivity	Ref
4G8	Raised against synthetic peptide Aβ17-24; epitope lies within aa 18–23; recognises multiple forms of Aβ	Cross reacts with APP770 and P3; reacts with conformational epitope of aggregated fibrils including α-synuclein	[2–4]
6E10	Raised against Aβ1-17; epitope lies within aa 4–9; recognises Aβ with intact N-terminal epitope	Cross reacts with APP and Aβ(1–16); No reaction predicted with P3	[2, 5]
6F3D	Raised against synthetic peptide Aβ8-17; epitope lies within aa 10–15; recognises Aβ with intact N-terminal epitope	Predicted to react with Aβ(1–16); Does not react with P3	[2, 6]
MBC40 (Aβ40)	Recognises C-terminal Aβ peptides ending at aa40; epitope not well described	Cross reacts with N-terminal truncated peptides including P3	[2]
MBC42 (Aβ42)	Recognises C-terminal Aβ peptides ending at aa42; epitope not well described	Cross reacts with N-terminal truncated peptides including P3	[2]
BS85	Raised against Aβ(25–35); recognises Aβ38, Aβ39, Aβ40, Aβ42 and Aβ43; epitope not well described	Cross reacts with N-terminal truncated peptides including P3	[2]
BC05	Raised against Aβ(35–43); recognises Aβ42 and Aβ43; epitope not well described	Cross reacts with N-terminal truncated peptides including P3; does not recognise Aβ40; used in commercial ELISA kits for the detection of Aβ42	[7, 8]
BA27	Raised against Aβ(1–40) Recognises Aβ40; 100-1000x more reactive with Aβ40 than Aβ42 and Aβ43; epitope not well described	Cross reacts with N-terminal truncated peptides including P3; used in commercial ELISA kits for the detection of Aβ40	[7]
AβN17 (Leu)	Raised against P3(40); recognises P3(40) and synthetic P3(42) peptide; epitope not well described	Reactivity with insoluble, aggregated P3(42) not confirmed	[7, 9, 10]
3D6	Raised against Aβ with N-terminal aspartic acid; epitope lies within aa 1–5; recognises multiple C-terminal variations	Does not cross react with sAPPs or full length APP; No reactivity with N-terminally altered Aβ; No reaction predicted with P3; parent of Bapineuzumab	[11–13]
266	Raised against synthetic Aβ; epitope lies within aa 13–28; recognises soluble monomer and multiple C-terminal variations	Cross reacts with various plasma proteins containing the core sequence KLVFF; does not cross react with P3; parent of Solanezumab	[11–14]

An updated version of Table 1 is published in this erratum, with the inclusion of three new references [12–14].

This evidence do not in any way undermine the argument that the cross-reactivities of anti-amyloid antibodies may confound research, and in fact can be interpreted as strengthening the argument.

The cross-reactivity of both Bapineuzumab and Solanezumab with various Aβ C-terminals and the cross reactivity of Solanezumab with various plasma proteins does not clarify the understanding of the APP proteolytic system and its role in disease, or identify with any certainty which peptides are of interest and are being targeted.
